# Reports of Societies

**Published:** 1876-08

**Authors:** 


### Reports of Societies.

### EIGHTH ANNUAL MEETING OF THE ASSOCIATION OF AMERICAN MEDICAL EDITORS.

(F. H. Davis, Secretary.)

The members of the Association assembled in the parlor of the Centennial Hotel, in Philadelphia, on Monday, May 5, 1876.

The meeting was called to order by the president, Dr. A. N. Bell, of New York. The following members were present: Drs. Theophilus Parvin, of Ind.; Leartus Connor, of Detroit; A. C. Garrett, of Boston; Robt. Battey, of Atlanta; H. Z. Gill, of St. Louis; W. H. Byford, of Chicago; H. C. Wood, of Philadelphia; L. D. Bulkley, of New York; D. G. Brinton, of Philadelphia; W. T. Briggs, of Nashville; Thos. S. Powell, of Southern Medical Record, and A. N. Bell, of Brooklyn.

The secretary, Dr. F. H. Davis, of Chicago, read the minutes of previous meeting, which were accepted and approved. The president then delivered the annual address; this was devoted mainly to an able and eloquent appeal for a higher standard of requirement for admission to the profession of medicine in this country. The facilities offered by most of our medical colleges were abundant; but the time of attendance required of the student, two terms, of four to six months each, was absurdly insufficient to enable him to intelligently profit by the advantages offered. Dr. H. C. Wood fully endorsed the views advanced in the president's address. In the early days of medical instruction in this country the colleges attempted to teach only the fundamental branches, and those they taught well and thoroughly. Now the schools attempt to cover the whole vast field of modern medical sciences, including the various specialties, and still expect the student to comprehend all of this in the old four or six months session. The result was that the graduates of to-day went out with a superficial smattering of all, but really thoroughly grounded in no one department. He believed in supporting Harvard and such schools as had the courage to come bodly forward and institute the reforms of a three year's graded course; uphold these institutions until their success should compel the others to follow.

Dr. Byford counseled moderation. He thought that most of the colleges were doing very well, as well as they could. Give them time enough and they would accomplish all the needed reforms. In reference to the deficiency of preliminary education on the part of medical students, he thought that the practitioners who sent these imperfectly prepared students to the colleges, were in the main responsible. Let the colleges have better material and they would turn out better educated physicians.

Dr. Palmer stated that in introducing this requirement of preliminary examination into Michigan University, they had found it necessary to reject several applicants. The preceptors of these gentlemen were very much offended, and the students easily gained admission at other schools where no such preliminary acquirements were necessary.

The question of State laws regulating the practice of medicine was discussed at some length by several members. Dr. Connor, of Detroit, said that he had taken opportunity to examine somewhat carefully the effects of the laws now in force in Canada, which require each practitioner of medicine to pass examination by a mixed board, containing representatives of the different schools of medicine, homœopathic, eclectic, etc. The result, he stated, had been that those districts were overrun with a class of unprincipled quacks and charlatans who possessed sufficient knowledge to pass the examining board, and were thus licensed to practice, and could compel recognition and admission to all medical societies, while they were under no moral or ethicical restraints. Their advertisements and posters were to be seen on every lamp-post and fence corner throughout the country.

It was agreed by the members of the Association generally that the profession must establish and maintain its own requirements for admission to its ranks, and its own ethics; also that some standard of acquirement, other than the possession of a simple college diploma, should be required for admission to the State Medical Societies and the American Medical Association.

The following resolution was offered and unanimously adopted:

*Resolved*, That we express our approval of such medical schools as require preliminary examinations and a three year's graded course of instruction, with stated examinations.

The Association then proceeded to the election of officers, with the following result: President, Dr. H. C. Wood, of Philadelphia; permanent secretary, Dr. F. H. Davis, of Chicago.

The meeting then adjourned to the evening preceding the next annual meeting of the American Medical Association.

---

### PROCEEDINGS OF THE ASSOCIATION OF THE REPRESENTATIVES OF AMERICAN MEDICAL COLLEGES, HELD AT PHILADELPHIA, JUNE 2 AND 3, 1876.

A convention of representative of numerous medical colleges of the United States was held in the hall of the Jefferson Medical College, of Philadelphia, June 2 and 3, 1876, in pursuance of the following call:

LOUISVILLE, KY., May 15, 1876.

Following a general correspondence with the various medical colleges of the United States, the undersigned issue this call for a convention, to be held in Philadelphia, on Friday, June 2, 1876, four days in advance of the meeting of the American Medical Association. The object of the convention is to consider all matters relating to reform in medical college work.

That decided results may be reached, the faculty of each college is requested to send one or more delegates, clothed with *plenary* powers to determine final action on every question.

Should any college find it impracticable to send a representative, it is hoped that it will set forth fully, by letter to the convention, the views it may hold touching the suppression of existing evils and methods of practical improvement.

Officers of the following colleges have informally signified their hearty approval of the movement:

Jefferson Medical College, College of Physicians and Surgeons, N. Y., Bellevue Hospital Medical College, Ohio Medical College, Miami Medical College, Rush Medical College, Detroit Medical College, Louisville Hospital Medical College, Medical Department of University of Louisville, St. Louis Medical College, Keokuk Medical College, Cleveland Medical College, Starling Medical College, Medical Department of Georgetown College, Medical Department of Columbian University. Long Island College Hospital, Medical Department of Syracuse University, Evansville Medical College, Indiana Medical College, Medical Department of University of Nashville, Atlanta Medical College, Mobile Medical College, Savannah Medical College, Augusta Medical College.

The convention will be called to order in the hall of the Jefferson Medical College, at eleven o'clock A. M., on the day above named.

J. B. Biddle, M. D., Jefferson Medical College.
Wm. H. Mussey, M. D., Miami Medical College.
John T. Hodgen, M. D., St. Louis Medical College.
J. Adams Allen, M. D., Rush Medical College.
W. T. Briggs, M. D., Med. Dep't Univ'ty of Nashville.
J. M. Bodine, M. D., Med. Dep't Univ'ty of Louisville.

At the hour named the following representatives assembled;

Jefferson Medical College—Prof. J. B. Biddle and Prof. S. D. Gross.

Medical Department University of Pensylvania—Prof. R. E. Rogers.

College Physicians and Surgeons of New York—Prof. Edward Curtis.

Medical Department University of Louisville—Prof. L. P. Yandell, Jr., and Prof. J. M. Bodine.

Hospital College of Medicine of Louisville—Prof. J. A. Larrabee and Prof. T. C. Wilson.

Long Island Hospital Medical College—Prof. J. H. Raymond.

Medical Department University of Iowa—Prof. E. Clapp.

College of Physicians and Surgeons, Syracuse University—Prof. H. B. Wilbur and Prof. Van Dyne.

Chicago Medical College—Prof. L. Curtis.

Medical Department University of Georgia—Prof. E. Geddings.

Indiana Medical College—Prof. T. B. Harvey and Prof. L. D. Waterman.

Medical Department University of Wooster—Prof. W. J. Scott.

Cleveland Medical College—Prof. J. H. Bennett and Prof. Heims.

Detroit Medical College—Prof. E. W. Jenks and Prof. L. Connor.

Starling Medical College—Prof. S. Loving.

Medical Department University of Vermont—Prof. H. D. Holton.

St. Louis Medical College—Prof. J. L. B. Alleyne.

Atlanta Medical College—Prof. W. F. Westmoreland.

Medical Department University of Nashville—Prof. W. T. Briggs.

Medical Department Vanderbilt University—Prof. T. A. Atchison.

Missouri Medical College—Prof. A. P. Lankford.

Keokuk College Physicians and Surgeons—Prof. J. J. M. Angier.

Columbus Medical College—Prof. J. F. Baldwin.

On motion of Prof. Yandell, Prof. J. B. Biddle was elected president of the convention, and on motion of Prof. Bennett, Prof. Leartus Connor was elected secretary.

On motion of Prof. E. Curtis, it was

*Resolved*, That the action of the convention shall not be considered binding upon the colleges represented unless endorsed by their respective faculties.

On motion of Prof. Gross, it was

*Resolved*, That a committee be appointed to submit business for the consideration of the convention, to report at the afternoon session.

The chair appointed as this committee, Profs. Bodine, Gross, Geddings, Holton and Scott.

The convention adjourned until four P. M.

Pursuant to adjournment the convention re-assembled at four P. M., the president in the chair.

The minutes of the last meeting were read and approved.

Prof. Bodine, from the committee to prepare business for the convention, reported the following questions for its consideration:

1st. Shall the beneficiary system, with its present abuses, be condemned or endorsed?

After discussion, on motion of Prof. E. Curtis, the following preamble and resolutions were adopted with reference to question first:

WHEREAS, The practice of reducing or remitting in individual cases the established fees of a college has the objectionable feature of discriminating between students who may be equally deserving, and opening the door to possible gross abuses; therefore

*Resolved*, *first*, That this convention regards the above privilege as one to be deprecated in general, and, if put into practice at all, to be exercised both rarely and reluctantly, and only in unusual circumstances, and after unsolicited application by proven deserving candidates.

*Resolved*, *second*, That anything like a wholesale system of such reduction or remission of established fees, or any open solicitation of recipients of such favors be regarded as in the highest degree improper, and that any college indulging in such practices deserves to forfeit its place on the *ad eundem* list of medical colleges.

2d. Shall two consecutive courses of lectures in one year entitle students to become candidates for garduation?

On motion of Prof. E. Curtis, it was

*Resolved*, That it is the opinion of this convention that no two consecutive sets of lecture tickets shall be regarded as fulfilling the usual prerequisites of instruction for graduation, where the time between the beginning of the first course and the end of the second is less than fifteen months.

3d. Shall any faculty under any circumstances issue a diploma not bearing the graduate's name?

On motion of Prof. Waterman, it was

*Resolved*, That no medical faculty should issue a diploma not bearing the graduate's name.

It was ordered that the meetings of the convention shall be at ten A. M. and four P. M. On motion the convention adjourned.

The convention reassembled on Saturday, June 3, at ten A. M., the president in the chair.

The minutes of the previous meeting were read and approved.

On motion of Prof. L. P. Yandell, Jr., the regular order of business was suspended, and communications were read from the faculties of the following medical colleges: Louisville Medical College, Kentucky School of Medicine, Evansville Medical College, Rush Medical College, Medical Department University Louisiana, Medical School of Harvard University, Savannah Medical College, Cincinnati College of Medicine and Surgery, Medical College of State of South Carolina.

On motion of Prof. Atchison, these communications were placed on file.

4th. Shall this convention resolve itself into a permanent organization?

On motion of Prof. Atchison, it was

*Resolved*, That the question be referred to a committee of five, to report at the afternoon session.

The chair appointed as this committee Profs. Atchison, L. Curtis, E. Curtis, Yandell and Scott.

On motion of Prof. Rogers, the president and secretary of the convention and Prof. Atchison were appointed a committee on publication.

5th. Is there any reason why the customary diploma fee shall be abolished ?

On motion of Prof. Rogers, it was

*Resolved*, That it is the sense of the convention that the diploma fee should not be abolished?

6th. Is it advisable to adopt a graded course of study?

On motion of Prof. Bodine, the following preamble and resolution were adopted in reference to this question.

WHEREAS, A knowledge of the elementary branches of medicine should precede a study of the practical branches,

*Resolved*, That, in the hope of inducing students to prolong and systematize their studies, this convention recommends to all medical colleges to offer to students the option of three courses of lectures, after a plan similar to the following: Students who have attended two full courses of lectures on anatomy, chemistry, materia medica and physiology, may be examined upon any of these subjects at the end of their second course. During their third course such students may devote themselves to the lectures upon the theory and practice of medicine, surgery, obstetrics and diseases of women and children, upon which subjects only they shall be examined at the final examination for the degree of M. D.—their standing however to be determined by the results of both examinations.

On motion, adjourned till four P. M.

The convention re-assembled at four P. M., the president in the chair.

The minutes of the last meeting were read and approved.

Prof. Atchison, from the committee to whom the subject of permanent organization was referred, reported the following resolutions:

*Resolved*, *first*, That this *convention* now proceed to form a Provisional Association of American Medical Colleges, under its present officers.

*Resolved*, *second*, That when the Association adjourns, it shall adjourn to meet at the call of its president.

*Resolved*, *third*, That the various medical colleges be invited to take into consideration the project of forming, at the next meeting of this Provisional Association, a Permanent Association of American Medical Colleges.

*Resolved*, *fourth*, That for the furtherance of this object a committee of three be appointed at this meeting to confer by letter with the various colleges, and invite their views on the proper object and plan of such proposed organization; and upon the receiptof the same, to draft a constitution and by-laws for a Permanent Association, to be submitted at the next meeting of this Association.

*Resolved*, *fifth*, That the advisory resolutions upon matters of college policy passed by this convention be printed and forwarded to all regular medical colleges in the United States for their consideration.

The chair appointed as committee to carry out the foregoing resolutions Prof. T. A. Atchison, Prof. Edward Curtis and Prof. L. P. Yandell, Jr.

These resolutions were adopted, and the convention resolved itself into the Provisional Association of American Medical Colleges.

7th. Is it proper for a regular college to have any kind of alliance with homœopathy?

On motion of Prof. Atchison, it was unanimously

*Resolved*, That in the opinion of this Association, medical colleges ought not to recognize or hold fellowship with any school or its alumni in which irregular medicine is taught as a part of the curriculum.

8th. Can college fees be made uniform?

On motion of Prof. Geddings, this question was referred to a committee of five, to report at the meeting of the Association to be held in 1877.

The chair appointed Profs. Geddings, Gross, Angier, E. Curtis and L. Curtis, this committee.

On motion of Prof. Biddle the following resolution was unanimously adopted:

No degree in medicine should be conferred under any circumstances, except after an examination in person of the candidate upon all the branches of medicine.

On motion of Prof. Atchison, the thanks of the Association were tendered to the president for the able and impartial manner in which he had discharged the duties of the chair.

On motion of Prof. Yandell, the thanks of the Association were tendered to the secretary for his efficient services.

On motion of Prof. Larrabee, the thanks of the Association were tendered to Jefferson Medical College for the use of the hall, and other courtesies.

On motion, the Association adjourned to meet at the call of the president.

J. B. BIDDLE, M. D. *President.*

LEARTUS CONNOR, M. D., *Secretary.*

### CHICAGO SOCIETY OF PHYSICIANS AND SURGEONS.

*April* 24, 1876.

The following paper was read by Dr. Hyde, on the use of salicylic acid in inflammatory rheumatism:

### Case I. Polyarthritis Rheumatica.

(Treated by Dr. MacArthur.)

W. N. B., aged twenty-nine, was the subject of an attack of inflammatory rheumatism precisely three years ago, affecting all the joints, and complicated with endocarditis, lasting four weeks.

March 1st. Found patient in bed with several of the larger joints affected, very emaciated and great debility from riotous living and dissipation. Was immediately put on the alkaline treatment with iodine applications to the swollen joints and rolled up in cotton batting. This was continued, with the addition of tonics, nourishing diet, etc., for fifteen days, my patient becoming weaker and the rheumatic difficulty growing worse from day to day.

March 16th. Patient very much depressed in spirits, haggard countenance, anemic murmur at base of heart and every joint in body affected with rheumatism. All previous treatment discontinued; was ordered 10 gr. doses of salicylic acid every two hours in wafer paper at 10 A. M.

Met Prof. Allen at 7 P. M. in consultation. Found patient very much improved; countenance quite bright, with the stiffness and soreness of joints very much diminished. Prof. Allen very kindly consented to give the salicylic acid a fair trial, although he had no faith in it.

17th. Patient exclaimed, on my entry into the room, that he had no use for me, his rheumatism was all gone; had slept well for the first time since his illness began; except when awoke to take his medicine, slept continually all night. Had only taken 120 grs. of the acid, producing no unpleasant effects, except hyperæsthesia in hearing. On account of illness, did not visit patient for five days, when I learned that the day prior he had some rheumatism in his left shoulder, elbow and wrist. Ordered the acid to be repeated, when he now took 240 grs. with entire removal of all rheumatic symptoms from this time forward; no relapse occurred, and his recovery was rapid and permanent. The treatment followed is that of Dr. Stricker, of Charity Hospital, Berlin.

Case II. Acute inflammatory rheumatism cured in 36 hours; 360 grs. of the acid administered in 10 gr. doses consecutively.

M. B. Thompson, aged forty-six, came to Chicago last fall from Alabama, where he had been a victim of yellow fever and intermittent; had the last disease for the past three years whilst living south.

March 26th. Arrived at patient's house at 4 P. M.; found him unable to stir hand or foot, or turn over in bed; face flushed, temperature 104.5, pulse 136, respiration 20, tongue coated; was suffering most acute pain in all his joints, which were very tender and swollen; even the carpal and phalangeal articulations were involved. I immediately prescribed the acid in 10 gr. doses, to be taken every two hours; no other medicine.

27th, 10 A. M. Temperature 101°, pulse 97, slept well after 2 A. M.; joints could be moved freely without producing much pain; swelling diminished, tongue moist, perspiring freely; the acid to be continued.

28th. Pulse 80, temperature 99°; swelling of joints entirely disappeared; could be freely handled and moved about without producing the slightest pain. Ordered the acid to be continued every four hours. This was my third and last visit, as patient's wife reported from day to day his condition. The acid was continued until he had taken 360 grs. in all, when the rheumatic symptoms had all subsided. On the following day—the fifth of the disease—was put on a tonic of quinine and iron; convalescence was rapid; walked around the house on the sixth day, and continues to improve daily. No toxic effects from the drug except ringing in his ears and somewhat dull in hearing.

### Case III. Rheumatism Treated by Salicylic Acid.

(Case treated by Dr. A. Fisher.)

G., an Italian, fifty years of age, had suffered from previous attacks of rheumatism. On March 13, 1876, Dr. Fisher was called to see him in a close and poorly ventilated apartment in the third story of a public building. He was then found to be suffering from a rheumatic affection of the wrists and knees, which were swollen to such a degree as to produce immobility; quite painful and tender, and exhibited externally a roseate flush. There was also fever, a rapid pulse, and the tongue coated with a thick pasty secretion. The patient improved under the treatment by colchicum and the iodide of potassium until three or four days had elapsed, when all the symptoms recurred with the severity of the first manifestations of the disease. On the 22d of March he was placed on 10 grains of salicylic acid given every two hours, night and day, until two drachms had been taken.

March 23d. The patient declared he was cured; the swelling of the joints, pain and tenderness had quite subsided. The acid was given every four hours only.

March 24th. Improvement rapid and satisfactory. Tongue clean and appetite returning. Ordered the acid to be given for a brief time thrice daily and discontinued attendance.

---

### THE CHICAGO MEDICAL SOCIETY.

*Meeting, May* 15, 1876.

(Reported by J. Suydam Knox, M. D.)

Prof. Jos. P. Ross exhibited a pathological specimen of tumor of the brain, with its history, as furnished by Dr. Cunningham, of the Cook County Insane Asylum. The following is a brief summary:

Peter Riley, Irish laborer, aged thirty-one, was admitted to the asylum Oct. 21, 1873, as a case of mania epileptica, and died there in convulsion Feb. 15, 1876. From three months previous to admission in the asylum, until death, the patient was subject to apparent epilepsy, falling suddenly convulsed and unconscious, without premonitory symptoms, and recovering from the attacks without stupor. These convulsions occurred from once to three times a day, with occasional intermissions of three or four days. His gait was shuffling and staggering, not favoring either side. His speech was thick and incoherent, and his intellect impaired, with inability to converse or reason. He however daily recognized his attending physician. He walked about the ward the day before his death.

An autopsy revealed the following abnormal conditions. The vessels of the meninges were extensively engorged with blood, and the parietal grooves for the middle meningeal arteries were extremely well marked. Imbedded in the substance of the left convolution of the longitudinal fissure, near its upper margin posteriorly, and entirely unconnected with the meninges, was a calcareous formation of irregular shape, about one inch in length, $\frac{3}{4}$ inch in breadth at widest part, and $\frac{1}{4}$ inch in thickness. Separated from this growth and close to it was another of similar character, about twice the size of a pea. Directly above this spot, in the convolution, the arachnoid was slightly covered by a bluish white, purulent appearing substance, and the dura mater was more than normally adherent to the parietal bone. The left lobe of the cerebellum was almost entirely wasted, what remained being so disorganized as to break down under the slightest touch. There was no indication of any previous injury of the skull.

Dr. Ross expressing regret at the meagre history of the case, reviewed briefly the tumors of the brain, viz.: 1. Those connected with the meninges of the brain and its cavities—tubercular and syphilitic. 2. Those associated with the sheaths of bloodvessels—tubercular, syphilitic and cancerous. 3. Those arising from the neuroglia of the brain itself—called by Virchow gliomata. The Dr. then put the question whether the specimens presented were the ultimate product of a tubercular tumor, with subsequent calcareous degeneration, or a similar degeneration of a glioma.

Dr. H. M. Lyman thought that the specimens were the ultimate product of a glioma with subsequent ossification. The convulsions, from their character, were epileptiform and not epileptic.

Dr. E. L. Holmes expressed the opinion that the tendency of gliomata arising from the retina was to increase and absorb surrounding tissues, not to degenerate. Tumors any where in the brain very often produce blindness. It is extremely difficult to locate them, with blindness as the prominent symptom. He then briefly narrated a case (reported in Archives of Ophthalmology and Otology, Vol. 4, 1875,) occurring in his practice several years ago. The patient first experienced a fullness of the head with complete deafness in the left ear, soon followed by a peculiar intra-cranial sound syrchronous with the pulse. Gradually vision of the left eye began to fail and then that of the right. Soon after the sense of smell became totally, and the sense of taste partially destroyed. The patient also experienced the most intense and disagreeable subjective odor, also at times agonizing pains in the right hip joint. In the last months of the illness there was progressive muscular paresis, with extreme emaciation. The patient died after a three years' illness, with all the special senses destroyed except hearing in the right ear. The mental faculties and the power of speech remained until the last two days. An autopsy revealed a tumor as large as a hen's egg at the base of the brain, which proved to be a hypertrophied pituitary body. The tumor had pressed upon the carotid artery, causing a large anærism. A quantity of serum was found in the ventricles so large as apparently to have absorbed all the white substance of the brain.

Dr. W. F. Lewis next reported a most interesting case of pleuro-pneumonia, with ulceration of the lung and pleuræ and escape of air into the cellular tissue. The Dr.'s history of the case was as follows:

"I was called to attend Mr. G. on the 30th March, 1876, the third day of his illness. There was great dyspnœa, the patient being compelled to sit up in bed; pulse 110; respiration 30; temperature normal; skin moist; abdominal breathing. There was crepitation of air in the cellular tissue over the whole chest and a part of the neck, with considerable tumefaction and fullness of the right side of the chest. Physical exploration of the chest gave on palpation vocal fremitus, exaggerated over left and absent over right lung, save at the apex; on percussion, dullness over entire right lung except at the apex, which with the left was tympanitic. Auscultation revealed tubular breathing over the entire right lung, except at the apex, where there was exaggerated respiratory sound. Patient stated he was taken sick March 27th with acute pain in right side, and said 'something had given way there.' There then occurred slight chill, attempts at vomiting, dry hacking cough, with 'swelling of the neck,' which increased by nearly one-third its natural size; this lasting, however, only for a short time."

There was no history of hereditary disease, and patient was apparently a robust man, though for a number of months he had been slowly failing in health. Death occurred April 1, 1876, after an illness of five days. Autopsy made the following afternoon. By special request of the friends thorax alone opened. Body well nourished. Enlargement of right side of chest very noticeable, also the presence of air in the subdermal cellular tissue. Upon removal of the sternum there was a large escape of serum from the right thoracic cavity, turbid with flakes of lymph and with pus. The lower two-thirds of the lung was bound in many places to the costal pleura by firm adhesions, especially so over the middle anterior surface of the lower lobe. These adhesions were so firm that it was impossible to remove the lung in fit condition for proper examination. The upper half of superior lobe of right lung, and the whole of left lung, appeared normal. The balance of the right lung was hepatized. At about the middle of the anterior surface of the lower lobe, (the point where the adhesion to the costal pleura was the densest,) a cavity was found filled with pus.

Dr. J. P. Ross remarked that the case was undoubtedly one of pleuro-pneumonia, with adhesion of the pleuræ at the point indicated, subsequently the formation of a pulmonary cavity there, connecting with some large bronchial tube, and then ulceration through the adherent pleuræ and escape of air. He thought the case extremely rare; had never seen a similar one either in hospital or private practice. Pneumo-thorax, however, was common enough as a result of violence.

Dr. N. Bridge, who, in consultation, had seen the case the day before death, and had then observed the normal temperature, expressed some doubt as to whether pneumonia with hepatization of lung could have occurred so recently, and the temperature have returned so soon to the normal standard.

